# DELLA-Induced Early Transcriptional Changes during Etiolated Development in *Arabidopsis thaliana*

**DOI:** 10.1371/journal.pone.0023918

**Published:** 2011-08-31

**Authors:** Javier Gallego-Bartolomé, David Alabadí, Miguel A. Blázquez

**Affiliations:** Instituto de Biología Molecular y Celular de Plantas (CSIC-Universidad Politécnica de Valencia), Valencia, Spain; Ecole Normale Superieure, France

## Abstract

The hormones gibberellins (GAs) control a wide variety of processes in plants, including stress and developmental responses. This task largely relies on the activity of the DELLA proteins, nuclear-localized transcriptional regulators that do not seem to have DNA binding capacity. The identification of early target genes of DELLA action is key not only to understand how GAs regulate physiological responses, but also to get clues about the molecular mechanisms by which DELLAs regulate gene expression. Here, we have investigated the global, early transcriptional response triggered by the *Arabidopsis* DELLA protein GAI during skotomorphogenesis, a developmental program tightly regulated by GAs. Our results show that the induction of GAI activity has an almost immediate effect on gene expression. Although this transcriptional regulation is largely mediated by the PIFs and HY5 transcription factors based on target meta-analysis, additional evidence points to other transcription factors that would be directly involved in DELLA regulation of gene expression. First, we have identified *cis* elements recognized by Dofs and type-B ARRs among the sequences enriched in the promoters of GAI targets; and second, an enrichment in additional *cis* elements appeared when this analysis was extended to a dataset of early targets of the DELLA protein RGA: CArG boxes, bound by MADS-box proteins, and the E-box CACATG that links the activity of DELLAs to circadian transcriptional regulation. Finally, Gene Ontology analysis highlights the impact of DELLA regulation upon the homeostasis of the GA, auxin, and ethylene pathways, as well as upon pre-existing transcriptional networks.

## Introduction

Plants are sessile organisms that cannot change their location as a strategy to optimize their access to energy sources or in response to the environment. Thus, adjusting their growth and choosing the correct developmental program has to be precise and robust otherwise chances of survival could be reduced. This need has forced the development of very sophisticated sensing mechanisms and signal transduction pathways to respond properly to fluctuating environmental conditions. Plant hormones play an instructive role on this as they control many, if not all, developmental responses in plants [1], [2].

Gibberellins (GAs) are one of the classical plant hormones. They regulate several processes during the plant life cycle such as germination, vegetative growth or flowering [3] through gene transcriptional regulation [4], [5], [6], [7]. This transcriptional regulation relies on the activity of the nuclear, GA-regulated DELLA proteins [8]. In brief, DELLAs accumulate in the absence of GAs blocking the transcriptional response to the hormone. When GA levels increase, the binding of the hormone to its receptor, GID1, promotes the formation of a GA-GID1-DELLA complex [9], [10] that favors the recognition of the DELLA protein by the SCF^SLY^ ubiquitin ligase [11] and the subsequent ubiquitination. This modification leads to DELLA degradation by the 26S proteosome [12], [13] and transcriptional changes to the hormone take place.

Two observations support the idea that DELLAs are transcriptional regulators: first, chromatin immunoprecipitation (ChIP) experiments reveal that DELLAs sit at the vicinity of promoters of certain GA-regulated genes [6], [14]. Second, DELLAs interact physically with transcription factors and other transcriptional regulators. For example, they interact with bHLH transcription factors of the PIF clade and inhibit their ability to bind DNA [15], [16], as well as with other members of the bHLH family [17], [18]. Also, they interact with JAZ proteins, which are transcriptional regulators that negatively regulate jasmonate signaling [19], and with SCL3, a transcriptional regulator that belongs to the GRAS family [14], [20]. In addition, genetic evidence indicates that the bZIP transcription factor HY5 mediates the promotion of photomorphogenesis by DELLA [21].

Despite these recent advances, we still lack a broader view of the mechanisms by which DELLA proteins regulate the large variety of GA responses. A bottom-up strategy to dissect further this fundamental aspect of GA signaling is to identify and classify GA target genes according to their expression domain or the process in which they participate. In this regard, global analyses of DELLA-regulated transcription in two different developmental contexts –vegetative growth and floral development– have shown that only 3.6% of the target genes are shared between the two sets [4], [6]. This observation underscores the importance of the developmental context in which GA signaling is investigated.

GAs are important regulators of the skotomorphogenic developmental program [21], [22], [23]. In order to dissect how GAs regulate this process, we have searched for early target genes of DELLAs in etiolated seedlings. For that purpose, we have examined global, rapid changes in gene expression after compromising the GA signaling pathway in dark-grown seedlings. This approach allowed us 1) to identify which cellular pathways are directly regulated by GAs to promote skotomorphogenesis; and 2) to identify gene targets that will serve as markers to further dissect the mechanisms by which DELLAs regulate gene expression.

## Results and Discussion

### Identification of genes rapidly regulated by GAI in etiolated seedlings

**Figure 1:**
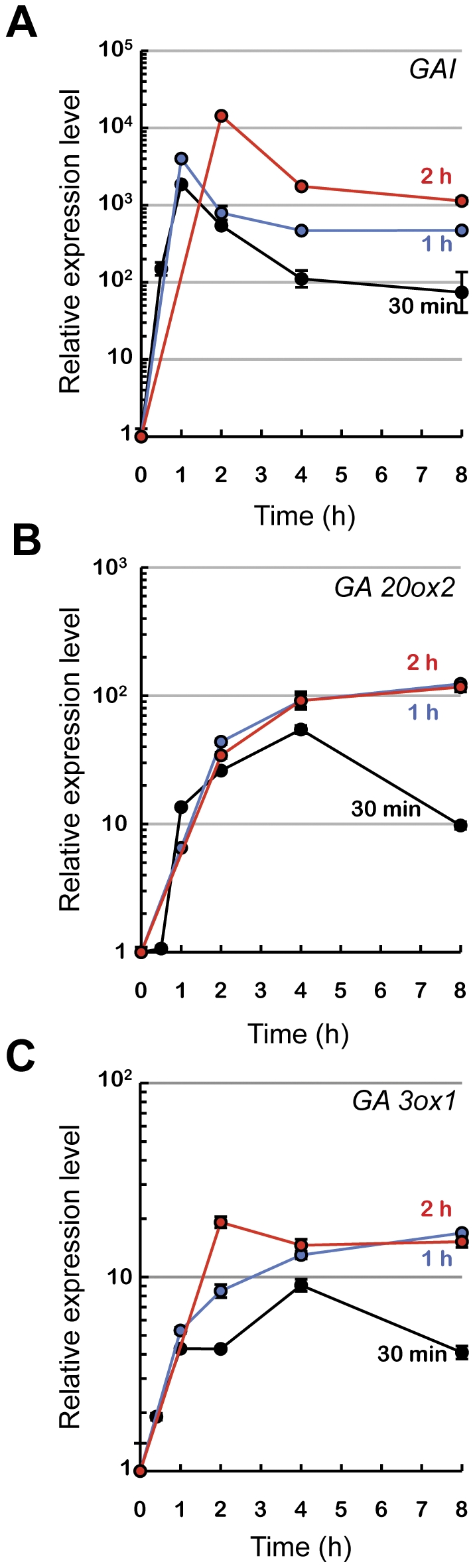
Effect of transient *gai-1* induction on known DELLA target genes. Two-day-old, etiolated *HS::gai-1* and wild type Col-0 plants grown at 22°C received a 37°C heat-shock treatment for different periods (30, 60, 120 min) and then returned to 22°C. Samples were collected at the indicated times. Expression of the transgene (A), as well as of *GA20ox2* (B) and *GA3ox1* (C) genes was monitored by RT-qPCR.

We sought to identify in a global and unbiased way genes whose expression was modulated rapidly in response to a change in GA activity in etiolated seedlings by using a transgenic line that expresses a gain-of-function version of the DELLA protein GAI under the control of a temperature-inducible promoter, *HS::gai-1* [21]. To determine the optimal duration of the heat treatment needed to strongly induce *gai-1* transcript accumulation, we placed 2-day-old etiolated *HS::gai-1* seedlings at 37°C for 30, 60, and 120 minutes, and then analyzed expression of the transgene by qRT-PCR over a time-course (Figure 1A). The 30-min treatment was sufficient to strongly and transiently induce *gai-1* transcript accumulation. To confirm that the inductive treatment resulted in an increase of GAI activity, we checked the expression of the *GA20ox2* and *GA3ox1* genes, that encode key enzymes in the GA biosynthetic pathway subject to feedback regulation by DELLA proteins [6], [24], [25], [26]. As expected, transcripts of both genes accumulated strongly in seedlings following the heat shock, but only in the 30-min treatment was this accumulation transitory (Figures 1B and C); moreover, expression of these genes did not change significantly in response to the temperature treatment in wild-type seedlings (data not shown).

**Figure 2:**
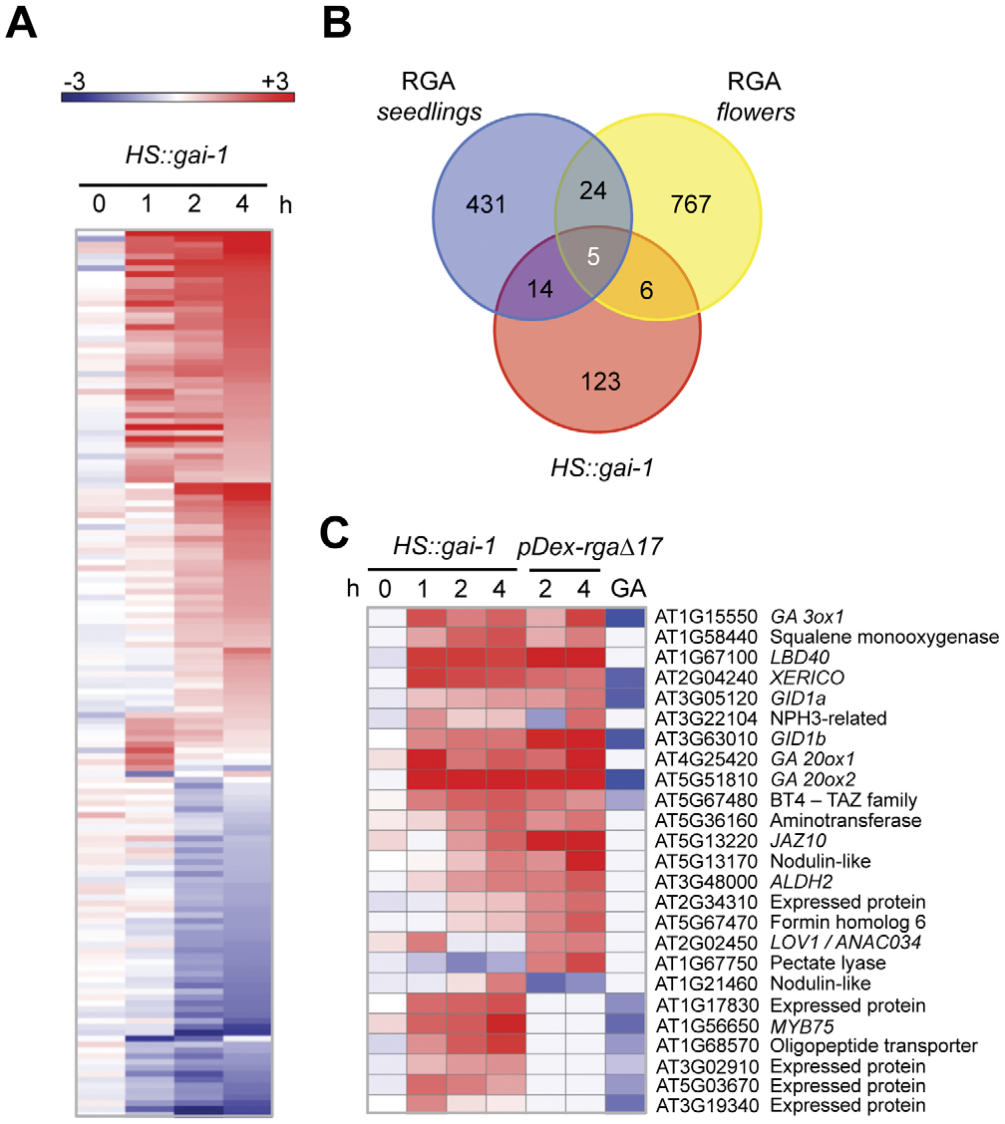
Transcriptomic analysis of early targets of DELLA proteins. (A) Heatmap representation of the 148 best-scored genes (q-value≤8). (B) Illustration of the overlap with the datasets of DELLA target genes in two other developmental situations [6], [73] (C) Heatmap representation of the differential expression of genes overlapping in the three datasets. Red and blue colors in the heatmaps represent induced and repressed genes, respectively.

Given that the induction protocol was appropriate to modulate the expression of GAI target genes, we interrogated the transcriptome of two-day-old etiolated *HS::gai-1* seedlings at 0, 1, 2, and 4 hours after starting a 30-min heat shock at 37°C. Expression was compared at each time point using triplicate RNA samples from whole transgenic seedlings and the corresponding wild-type seedlings by hybridization of 70-mer oligonucleotide, two-colors arrays representing the majority of the *Arabidopsis* genes (http://www.ag.arizona.edu/microarray). The microarray raw data have been deposited in the NCBI's GEO database under accession GSE24253. The application of a Significance Analysis of Microarrays criterion [27] with a false discovery rate of 8.74% and a 1.5-fold cutoff value allowed us to identify 148 genes differentially expressed during the first four hours after the induction of *gai-1* activity. This list represented the genes putatively regulated by GAI in etiolated seedlings (Table S1); among them, 58 were downregulated and 90 induced (Figure 2A).

Recently, a microarray analysis identified hundreds of genes whose expression is altered in the dark in the GA-deficient *ga1-3* mutant compared to the wild type [23]. Notably, only 18% of the GAI-regulated genes appeared equally misregulated in the *ga1-3* mutant (Figure S1). This little overlap is a likely consequence of the different experimental designs, aimed to investigate global gene expression in response to a short (this study) vs. a continuous blockage of the GA signaling pathway [23]. In addition, this clearly reflects the complexity of the dynamics of gene expression in response to DELLA proteins. For instance, the non-overlapping, GAI-regulated genes seem to respond only transiently since they were not misregulated in response to continuous accumulation of DELLAs. Conversely, the great majority of genes from the *ga1-3* experiment either was late responders or responded indirectly to DELLA accumulation. Importantly, this comparison highlights the suitability of our approach to identify early events downstream of the DELLA protein GAI in etiolated seedlings.

### Comparison of DELLA-regulated genes in different developmental situations

Recent studies have identified by a similar approach early target genes of the Arabidopsis DELLA protein RGA in aerial tissue of light-grown seedlings [6] and in flowers of Arabidopsis [4], as well as genes responding rapidly to GA application [6]. Despite the functional similarities between these two DELLA proteins [17], comparison of the sets of genes regulated by GAI and RGA showed little overlap. Out of the 148 GAI targets in etiolated seedlings, 19 genes overlapped with RGA targets in seedlings [6] and 11 in flowers [4], which corresponds to 13% and 7% of the GAI-regulated genes respectively (Figures 2B and C). Only five genes overlapped in all conditions (Figure 2B) and, remarkably, four of them encode members of the GA pathway (*GA20ox1*, *GA20ox2*, *GA3ox1*, and *GID1b*) supporting the notion that the strong regulation of GA activity by DELLA proteins extends to several tissues and growth conditions. However, beyond this regulatory process, a limited overlap in targets displayed by DELLA proteins is evident. It is unlikely that this effect is the consequence of the different expression patterns of the *DELLA* genes used in these studies, given that ubiquitous promoters were used to drive their expression [6], [21]. Rather, the low degree of overlap probably reflects the presence of very different sets of transcription factors available for DELLA interaction in etiolated seedlings (our current study) compared with light-grown seedlings and flowers.

### GAI regulates target genes in part through PIFs and HY5 transcription factors

**Figure 3:**
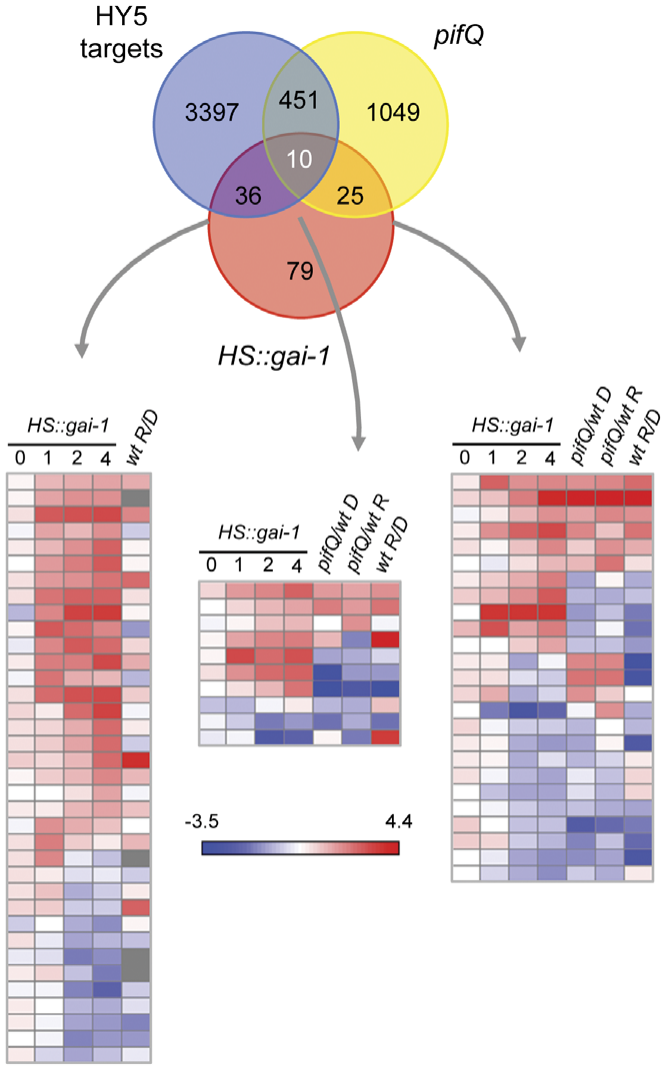
Meta-analysis comparing microarray data from *HS::gai-1*, HY5 and PIF targets. Venn diagram of microarray data from *HS::gai-1*, HY5 targets [30] and quadruple *pif* mutant (*pifQ*) [31] show common genes regulated by GAI, HY5 and PIF proteins. Heatmaps show the behavior of common GAI-HY5, GAI-HY5-PIF and GAI-PIF targets in different light conditions. **Wt R/D**, data are differentially expressed genes under red light compared to dark in a WT. **pifQ/wt D**, data are differentially expressed genes among *pifQ* mutant compared to wt in darkness. **pifQ/wt R**, data are differentially expressed genes among *pifQ* mutant compared to wt under red light. The heatmaps represent the differential expressions of genes overlapping in the different datasets. Red and blue colors in the heatmaps represent induced and repressed genes, respectively.

The proper control of the developmental switch between skotomorphogenesis and photomorphogenesis after germination is triggered by light through the activation of transcription factors that promote photomorphogenesis, like ELONGATED HYPOCOTYL5 (HY5), and the inactivation of other transcription factors that promote etiolated growth, such as the PHYTOCHROME-INTERACTING FACTORs, (PIFs) [28]. Remarkably, GAs counterbalance the effect of light by regulating negatively HY5 protein levels [21], and also alleviating the negative effect that DELLAs exert on the PIFs and that prevents the binding of these transcription factors to their target promoters [15], [16]. To investigate at the molecular level the extent of these functional interactions, we compared the list of GAI targets with the available lists of genes regulated by HY5 and the PIFs. We reasoned that this comparison would allow us to identify which GAI-regulated genes depend on the activity of these transcription factors, and delineate the transcriptional network that mediates the GA-control on this developmental switch. While a faithful dataset of *in vivo* target genes for HY5 in light-grown seedlings has been generated by ChIP-to-chip experiments [29], the only available list of putative PIF targets can be extracted from transcriptomic analyses of dark- and light-grown wild-type and *pifQ* mutants [30]. As shown in Figure 3, almost half of the GAI regulated targets are either regulated by HY5, the PIFs, or both, supporting the relevance of these transcription factors in transcriptional regulation by DELLAs.

The comparisons are consistent with current models of light and GA regulation. For instance, many of the genes whose promoters are bound by HY5 are coherently regulated by light treatments, and also by DELLA accumulation (Figure 3). Only a few of them displayed conflictive regulation by light and by DELLAs (induced by light, bound by HY5, repressed by DELLAs), probably indicating that these targets common to HY5 and DELLAs are not regulated jointly, but in parallel. In the case of PIFs, it is well established that DELLAs have a negative effect on PIFs activity [15], [16]. In agreement with this, many genes that are targets for both PIFs and DELLAs show the same behavior for DELLA accumulation and for PIF deficiency (Figure 3). An indication that this regulation is biologically relevant is that endodermis-specific expression of *PIF1* in *pifQ* mutants restores the formation of the apical hook [31], and this tissue specificity is also observed for the regulation of the apical hook by GAs [32]. But there are also some cases where the opposite behavior is observed, suggesting either that DELLA regulation of those targets does not proceed through PIFs, or that not all individual PIFs have equivalent activities and abilities to interact with DELLAs *in vivo*.

### Promoter analysis of GAI regulated targets suggests new transcription factors mediating DELLA activity

**Figure 4:**
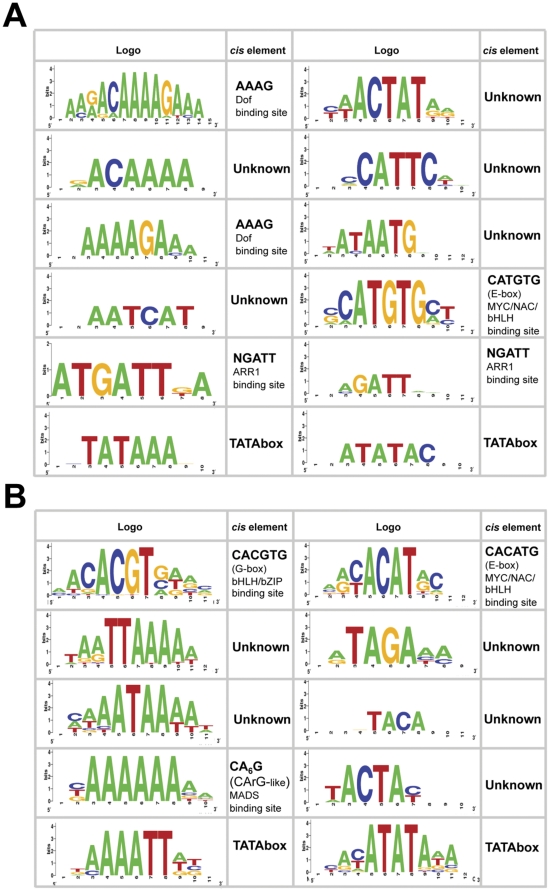
Over-represented *cis* elements among DELLA-regulated promoters. (A) Logos of over-represented *cis* elements in the promoters of induced and repressed targets in the *HS::gai-1* microarray experiment. (B) Logos of over-represented *cis* elements in the promoters of induced and repressed genes coming from the joint dataset of *HS::gai-1*, *rga-*Δ*17* [6] and GA/floral [4] microarray targets. The logo representation was obtained at http://weblogo.berkeley.edu/ [73].

Although half of the GAI targets are likely regulated by HY5 and PIFs, there is no obvious connection between these two transcription factors and the rest of the genes regulated by GAI. To get hints regarding the identity of the additional transcription factors mediating DELLA regulation, we investigated the enrichment of particular *cis* elements among the promoters of genes up- and downregulated in *HS::gai-1* using ELEMENT (http://element.cgrb.oregonstate.edu/) [33]. This tool returns those 3–8 bp sequences that are over-represented in the 1000 bp upstream region that precedes the transcription start site of target genes, compared to those same regions through the whole Arabidopsis genome. According to this analysis, apart from a small number of putative *cis* elements with unknown identity (Figure 4A), the promoters of genes induced by GAI are enriched in the Dof (AAAG) [34] and ARR1 (NGATT) [35] binding sites. Interestingly, both types of transcription factors have been related to GAs. For example, Dof proteins have been implicated in the regulation of GA signaling and biosynthesis in Arabidopsis and barley, possibly in the DELLA-mediated feedback regulation of the GA pathway [36], [37], [38]. And ARR1 has been shown to mediate the control of root meristem size in response to GAs through the up-regulation of *ARR1* expression by DELLA proteins [39].

To investigate if this analysis allows the identification of DELLA-related regulatory sequences common to different developmental contexts, we examined the enrichment of *cis* elements in the dataset containing all DELLA target promoters found in all available experiments [4], [6]. Surprisingly, the analysis showed an enrichment in two known regulatory sequences: the G-box (CACGTG) [40] and a sequence similar to the CArG box (CC(A/T)_6_GG) [41], which also includes a Dof binding site (AAAG) (Figure 4B). The presence of G-boxes is reasonable, taking into account that they are bound both by bHLH and bZIP transcription factors [29], [42], like the PIFs and HY5, for which strong molecular interactions exist with respect to GA signaling [15], [16], [21]. However, no link between MADS-box transcription factors and GAs has been established yet.

On the other hand, the E-box CATGTG also appeared as an over-represented sequence both in the etiolated and in the joint dataset of DELLA targets (Figure 4). E-boxes (CAnnTG) are usually bound by bHLH proteins. Unlike the G-box, which is a particular case of an E-box bound by PIFs [15], [16], [40], [43], the CATGTG (or CACATG, in the opposite orientation) is the E-box preferred for instance by the brassinosteroid signaling elements BZR1 and BES1 [39], [44], [45]. Moreover, this element is enriched in promoters of dawn-phased genes that oscillate under short-day photocycles, and it is important for gating their expression by the circadian clock [46]. Thus, the enrichment of this E-box element could subtend the connection between DELLA proteins and circadian regulation of transcription [26] or point to new interactions between the GA and brassinosteroid pathways.

### Identity of GAI-regulated genes

 To identify the basic biological processes that are regulated by GAs in etiolated seedlings at the molecular level, we followed two complementary approaches. In the first one, we searched for any significantly over-represented Gene Ontology term (GO) [47] in our gene list by using the FatiGO algorithm [48]. In the second approach, we paid attention to the appearance of annotations that could reveal suggestive connections between GA signaling and other signaling pathways. As expected, we found that GAI is closely involved in the control of GA homeostasis and growth, but we also found that GAI regulates the expression of genes directly implicated in light signaling, stress responses, transcriptional networks, and the synthesis and signaling of other hormones (Table 1).

**Table 1:** Gene Ontology (GO) categories statistically over-represented among DELLA targets.

BIOLOGICAL PROCCESS				MOLECULAR FUNCTION			
GO category	p-value	genes		GO category	p-value	genes	
Response to gibberellin stimulus	2.38E-09	*AT2G01570*	*RGA1*	oxidoreductase activity	5.95E-05	*AT4G25420*	*GA20OX1*
Gibberellic acid mediated signaling pathway	5.12E-08	*AT3G05120*	*GID1A*			*AT1G60980*	*ATGA20OX4*
Gibberellin biosynthetic process	1.23E-06	*AT2G37640*	*EXP3*			*AT4G21200*	*GA2OX8*
		*AT1G67100*	*LBD40*			*AT1G15550*	*GA3OX1*
		*AT1G66350*	*RGL1*			*AT5G51810*	*GA20OX2*
		*AT4G25420*	*GA20OX1*	transcription factor activity	1.66E-05	*AT5G56860*	*GNC*
		*AT5G25900*	*GA3*			*AT3G60390*	*HAT3*
		*AT3G63010*	*GID1B*			*AT1G49560*	*MYB TF*
		*AT2G04240*	*XERICO*			*AT4G00050*	*UNE10*
		*AT1G15550*	*GA3OX1*			*AT5G28300*	*trihelix DNA-binding*
		*AT5G51810*	*GA20OX2*			*AT1G56650*	*PAP1*
		*AT5G67480*	*BT4*			*AT3G50890*	*AtHB28*
Regulation of transcription	0.00485	*AT3G28857*	*PRE5*			*AT3G18010*	*WOX1*
		*AT4G39070*	*STH7*			*AT4G32280*	*IAA29*
		*AT1G66380*	*MYB114*			*AT1G53910*	*RAP2.12*
		*AT3G60390*	*HAT3*			*AT2G02450*	*ANAC035*
		*AT4G30180*	*bHLH146*			*AT2G42380*	*AtBZIP34*
		*AT1G49560*	*MYB TF*			*AT1G66380*	*MYB114*
		*AT4G00050*	*UNE10*			*AT4G39070*	*STH7*
		*AT5G28300*	*trihelix DNA-bind*			*AT1G69690*	*TCP TF*
		*AT1G53910*	*RAP2.12*			*AT3G06590*	*AIF2*
		*AT5G14750*	*ATMYB66*			*AT5G39860*	*PRE1*
		*AT1G14600*	*Myb-like TF*			*AT1G21910*	*AtERF012*
		*AT1G69690*	*TCP TF*			*AT2G01570*	*RGA1*
		*AT1G56650*	*PAP1*			*AT4G30180*	*bHLH146*
		*AT3G06590*	*AIF2*			*AT1G66350*	*RGL1*
		*AT5G15150*	*ATHB-3*			*AT3G15540*	*IAA19*
		*AT2G01570*	*RGA1*			*AT3G28730*	*ATHMG*
		*AT5G41920*	*SCL25*			*AT5G14750*	*ATMYB66*
		*AT4G32890*	*GATA9*			*AT1G14600*	*Myb-like TF*
		*AT1G21910*	*AtERF012*			*AT5G41920*	*SCL25*
response to red or far red light	0.000851	*AT2G01570*	*RGA1*			*AT5G15150*	*ATHB-3*
		*AT5G04190*	*PKS4*			*AT4G32890*	*GATA9*
		*AT2G37640*	*EXP3*	monooxigenase activity	0.00246	*AT5G25900*	*GA3*
		*AT4G32280*	*IAA29*			*AT4G28720*	*YUCCA8*
		*AT4G25260*	*invertase inhibitor*			*AT2G26710*	*BAS1*
		*AT1G15550*	*GA3OX1*			*AT1G58440*	*XF1*
		*AT5G51810*	*GA20OX2*			*AT5G38970*	*BR6OX1*
response to jasmonic acid stimilus	0.0193	*AT1G66350*	*RGL1*	lyase activity	0.0244	*AT3G51430*	*YLS2*
		*AT2G01570*	*RGA1*			*AT3G07010*	*pectate lyase*
		*AT1G66380*	*MYB114*			*AT1G27980*	*DPL1*
		*AT5G13220*	*JAZ10*			*AT1G67750*	*pectate lyase*
		*AT1G56650*	*PAP1*			*AT5G28020*	*CYSD2*
response to salt stress	0.0366	*AT1G13930*				*AT4G37770*	*ACS8*
		*AT2G01570*	*RGA1*			*AT5G36160*	*C-S lyase*
		*AT1G66350*	*RGL1*				
		*AT1G56650*	*PAP1*				
		*AT2G33380*	*RD20*				
		*AT2G04240*	*XERICO*				
unidimensional cell growth	0.0115	*AT5G51810*	*GA20OX2*				
		*AT4G25420*	*GA20OX1*				
		*AT2G37640*	*EXP3*				
		*AT2G20750*	*ATEXPB1*				
		*AT2G40610*	*ATEXPA8*				

### Direct regulation of the GA pathway by DELLA proteins

The control of the homeostasis of GA levels and perception in the plant is finely achieved through feedback and feedforward mechanisms that require the activity of the different elements of the GA signaling pathways [3], [49], [50]. Recently, Zentella et al. (2007) [6] demonstrated the involvement of the DELLA protein RGA in this process, as they showed that RGA directly up-regulates the expression of *GA20ox2*, *GA3ox1*, *GA INSENSITIVE DWARF1a* (*GID1a*), and *GID1b* genes. In addition to these genes, we have found *GA20ox1* and *GA20ox4* among the GAI up-regulated genes, and *GA2ox8*, *RGA*, and *RGL1* among the GAI down-regulated genes (Figure 2B, Table 1, and Table S1). The regulation of some of these genes by GAI was confirmed by analyzing their transcript levels in several GA-related mutants and transgenic lines (Figure S2). Control on the expression of the majority of genes seems to be shared by GAI, RGA, and also other DELLA proteins –for example, the repression of *GA2ox8* gene expression by PAC still occurs in the double null mutant *gai-t6 rga-24*.

**Figure 5:**
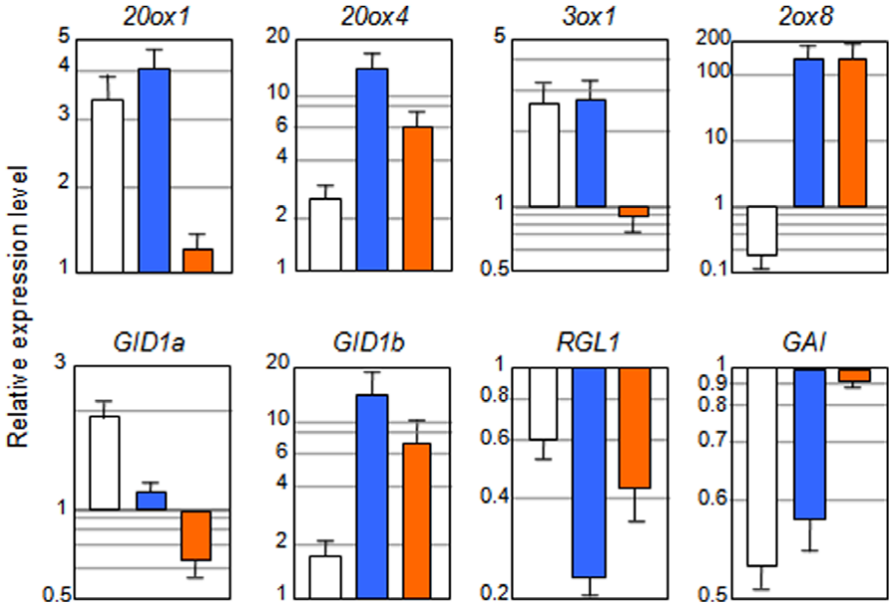
GAI directly regulates the expression of genes of the GA pathway. Three-day-old, etiolated *pGAI::gai-1-GR* seedlings grown at 22°C were incubated for 5 h in water or in water (control treatment) supplemented with either 10 αM DEX (white bars), 10 αM cycloheximide (orange bars) or both (blue bars). Expression was monitored by RT-qPCR and normalized to the control treatment. Values are log ratios between the treatment and the control. Data represent mean and the standard error of the mean from three independent biological replicates. Data from each biological replicate consisted in three technical replicates that were averaged and normalized.

 The rapid change in the expression of these genes in response to gai-1 accumulation suggested to us that they might be direct targets. We tested this possibility by using transgenic lines that express a translational fusion between *gai-1* and the glucocorticoid receptor domain from rats, under the control of the *GAI* promoter [51]. As expected, dexamethasone (DEX) treatment mimicked the effect on target gene expression that a heat-shock treatment provokes in the *HS::gai-1* line (Figure 5). Addition of cycloheximide (CHX) alone caused induction or repression of some target genes, suggesting that they are also regulated by short-lived repressors or activators, respectively. But most importantly, a clear induction of *GA20ox1*, *GA20ox4*, *GA3ox1*, *GID1a*, and *GID1b*, and a clear repression of *RGL1* and *GAI* was still observed in the simultaneous presence of DEX and CHX, indicating that these genes are directly regulated by GAI activity, i.e. independently of protein synthesis. It is difficult, however, to draw conclusions in the case of *GA2ox8*, given the strong upregulation of this gene in response to CHX. At first glance, results suggest that *GA2ox8* might not be directly regulated by GAI. However, the strong CHX effect could mask the repression exerted by GAI on this gene, as reported for *ACS8* that is a bona fide direct target [32].

Interestingly, the observation that GAI represses the expression of other *DELLA* genes is in agreement with a more general role for DELLAs controlling each other expression, and it provides a mechanism for the observation that *GAI* and *RGA* gene expression was higher in the presence of GAs [52].

### DELLA proteins mediate direct cross-regulation with auxin and ethylene pathways

Our analysis indicates that the crosstalk between GAs and other hormones could be exerted at the transcriptional level. Among the relevant targets for GAI, we identified several genes related to auxin synthesis and signaling, such as the negative auxin signaling intermediates *AUXIN/INDOLE-3-ACETIC ACID19* (*Aux/IAA19*) [53] and *Aux/IAA29*, two auxin-inducible *SMALL AUXIN UPREGULATED* genes, and also *INDOLE-3-ACETIC ACID METHYLTRANSFERASE1* (*IAMT1*) [54] and *YUCCA3* (*YUC3*) involved in IAA inactivation [55] and biosynthesis [56], respectively (Table 1 and Table S1). The ethylene biosynthesis genes *ACC SYNTHASE8* (*ACS8*) and *ACS5/ETO2* [57], [58] were also among the genes downregulated by GAI, extending the control by GAs to hormones other than auxin.

**Figure 6:**
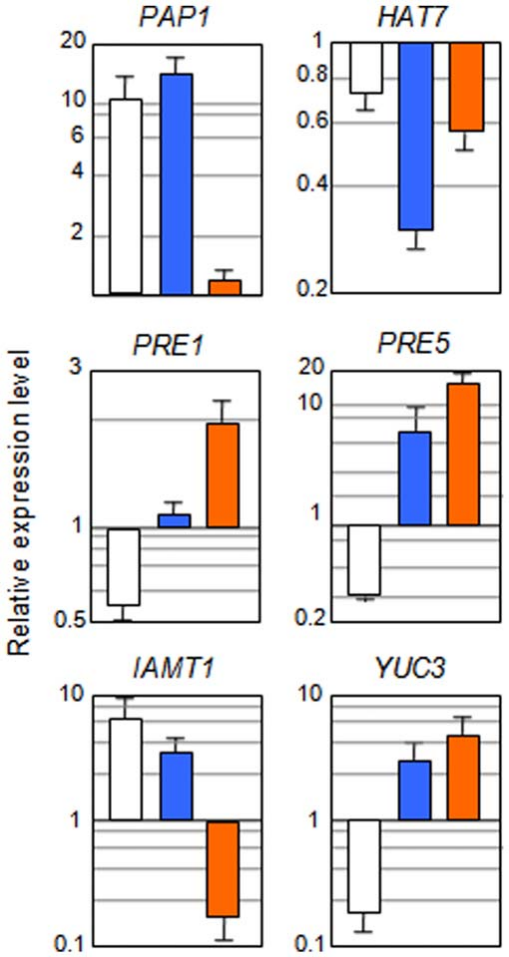
GAI directly modulates the auxin pathway and transcriptional networks. Three-day-old, etiolated *pGAI::gai-1-GR* seedlings grown at 22°C were incubated for 5 h in water or in water (control treatment) supplemented with either 10 αM DEX (white bars), 10 αM cycloheximide (orange bars) or both (blue bars). Expression was monitored by RT-qPCR and normalized to the control treatment. Values are log ratios between the treatment and the control. Data represent mean and standard error of the mean from three independent biological replicates. Data from each biological replicate consisted in three technical replicates that were averaged and normalized.

We analyzed if the expression of a representative set of these genes was directly regulated by GAI using the DEX system. Transcriptional control of *Aux/IAA19*, *IAMT1*, *YUC3*, and *ACS8* by GAI was direct, since CHX did not abolish the effect that DEX treatment had on their expression (Figures 6) [32], [51]. Other DELLA proteins, on the other hand, shared the control on the expression of these genes (Figure S3) [32], [51].

These results indicate the GA pathway may directly influence the metabolism and/or signaling cascades of other hormone pathways as a way to control different features of the skotomorphogenic developmental program. Some of these interactions have been proven biologically relevant. For instance, the control of *Aux/IAA19* expression by DELLAs modulates the intensity and the variance of the response to auxin, thereby conferring flexibility to tropic responses [51]. Similarly, downregulation of *ACS5/ETO2* and *ACS8* expression by GAI represents the mechanism for cross-regulation between GAs and ethylene during the development of the apical hook [32]. Further, the effect that the GA pathway might have on auxin metabolism through regulation of the *IAMT1* gene, adds a new layer of complexity to the web of interactions involving the cross-regulation of hormone metabolism [59].

### DELLAs impinge on transcriptional networks

 The enrichment of the GO term that defines transcription factors among the GAI targets indicates that the strategy by which GAs orchestrate the regulation of multiple cellular processes could be through the control of high rank regulators that in turn modulate subsets of the responses (Table 1). Several families of transcription factors were up- or downregulated by GAI, indicating no particular preference for structural features. By using the DEX system, we showed that the regulation of *PRODUCTION OF ANTHOCYANIN PIGMENT1* (*PAP1*), *HOMEOBOX-LEUCINE ZIPPER PROTEIN7* (*HAT7*), *PACLOBUTRAZOL RESISTANT1* (*PRE1*), and *PRE5* genes by GAI was direct (Figure 6). Moreover, this regulation was shared by other DELLA proteins (Figure S3).

Interestingly, some of the transcription factors are key regulators of processes in which GAs have been shown to be relevant. This is the case of *PAP1* , which encodes a *myb* transcription factor that simultaneously controls the expression of several steps in anthocyanin production [60]. Although the results involving GAs in the control of flavonoid production are contradictory and they largely depend on the tissue analyzed [61], [62], DELLAs are implicated in the promotion of anthocyanin accumulation [63], [64], and it is reasonable to think that this regulation occurs, at least in part, through PAP1.

In a similar way, the downregulation by GAI of *PRE1* and *PRE5*, that encode bHLH transcription factors that impair cell expansion [65], could link GAs with growth in certain circumstances, for example during skotomorphogenic development. PRE1 and PRE5 are HLH proteins that cannot bind DNA, and it has been shown that this type of transcriptional regulators exert their regulatory activity through physical interaction with other bHLH transcription factors for which the interaction is deleterious [66]. Therefore, the negative effect of DELLAs on *PRE1* and *PRE5* expression would indirectly affect the activity of additional transcriptional networks not identified in this analysis.

### Concluding remarks

 The enormous plasticity in plant development depends on highly wired, interconnected signaling networks that properly integrate endogenous and environmental cues [67]. In many cases, the cross-regulation between pathways occurs at the level of transcriptional regulation [68]. The output of the GA pathway largely relies on the activity of the transcriptional regulators DELLA proteins. Our transcriptomic analysis of DELLA responsive genes in etiolated seedlings reveals that the activity of the GA pathway directly influences other hormone pathways –ethylene and auxin– and pre-existing transcriptional networks. Furthermore, our results highlight that the comparison of DELLA target lists in different tissues and conditions, as well as the survey of enriched *cis* elements among the targets, is a promising strategy to understand at the molecular level the multiplicity in DELLA functions along plant development.

## Methods

### Plant material and growth conditions

*Arabidopsis thaliana* GA signaling dominant mutant *rga-Δ17* [25], the double loss-of-function *rga-24 gai-t6* [69] and *pGAI::gai-1-GR* [51] are in the L*er* background, while *HS::gai-1* and the *35S::gai-1* [21] are derived from Col-0 accession. Seeds were sterilized and stratified for 6 days in water at 4°C. Germination took place under white fluorescent light (90–100 µmol m^−2^ s^−1^) at 22°C for 6 h in a Percival growth chamber E-30B (http://www.percival-scientific.com). Seeds were plated in plates of half-strength MS medium with 0.8% (w/v) agar and 1% (w/v) sucrose supplemented with either 1 µM PAC or mock treatment and grown in darkness at 22°C for 3 days. For short-term treatments, seedlings were incubated in the dark in water supplemented with 10 µM CHX and/or 10 µM DEX. MS and PAC were from Duchefa (http://www.duchefa.com). DEX and CHX were from Sigma (http://www.sigmaaldrich.com).

### Real-time quantitative RT-PCR

RNA extraction, cDNA synthesis, quantitative RT-PCR (RT-qPCR), analysis, and primer sequences for amplification of *GA20ox2* and *EF1-*α genes, used to normalize all expression data, have been previously described [70]. RT-qPCR oligonucleotides sequences for the other target genes are listed in Table S2.

To analyze expression of transgenic *gai-1* in the *HS::gai-1* seedlings, we used an oligonucleotide annealing to the 5′ UTR of the *HSP18.2* gene, which is included in the construct, as the forward primer (5′-CCCGAAAAGCAACGAACAAT-3′), and an oligonucleotide annealing to the *gai-1* coding region as the reverse primer (5′-TCATTCATCATCATAGTCTTCTTATCTTGA-3′).

### Gene expression analysis by long oligonucleotide microarrays

Seeds of *Arabidopsis* Col-0 and *HS::gai-1* transgenic line were sterilized, sown, stratified, and germinated as described above. Seedling were grown for 3 days in darkness at 22°C. Then both wild type and transgenic seedlings were moved to 37°C for 30 minutes. After the heat-shock treatment plates were moved back to 22°C. Samples were collected at time points 0, 1, 2, and 4 hours after the beginning of the heat treatment. Three independent biological replicates were used for the analysis. Total RNA from whole seedlings was extracted as described above. RNA amplification, labeling, and hybridization of microarray slides were carried out as described [71]. Scanning of the slides, quantification of spots, and normalization were performed as previously described [72].

### Promoter analysis

Promoter analysis (http://element.cgrb.oregonstate.edu/) was done using the ELEMENT webtool (http://element.cgrb.oregonstate.edu/). Logos were built using the Weblogo webtool (http://weblogo.berkeley.edu/). The cluster lists are formulated by using the highest-count promoter core elements. All longer elements containing the core element are clustered together. PLACE database (http://www.dna.affrc.go.jp/PLACE/) was used to identify any known *cis*-acting element.

## Supporting Information

Figure S1 **Meta-analysis comparing microarray data from** ***HS::gai-1*** **and** ***ga1-3*** **seedlings.** Heatmap representation of the differential expression of genes overlapping between the *HS::gai-1* and the *ga1-3* datasets. Red and blue colors in the heatmaps represent induced and repressed genes, respectively.
(TIF)

Figure S2 **DELLA regulation of GA homeostasis.** The expression of genes of the GA pathway was monitored by RT-qPCR and normalized to the corresponding controls. Values are log ratios between the treatment and the control. PAC, fold change between 0.2 αM PAC- and mock-treated wild type L*er* seedlings; *gai1-ox*, fold change between transgenic and wild type Col-0 seedlings; *rga-*α*17*, fold change between *ProRGA:GFP-(rga-*α*17)* and wild type L*er* seedlings; *gai/rga* null M, fold change between *gai-t6 rga-24* and wild type L*er* seedlings; *gai/rga* null P, fold change between PAC-treated and mock-treated *gai-t6 rga-24* seedlings. Three-day-old, dark-grown seedlings of the different genotypes were used. Data represent mean and standard error of the mean from three independent biological replicates. Data from each biological replicate consisted in three technical replicates that were averaged and normalized.
(TIF)

Figure S3 **DELLAs regulate the expression of genes of the auxin metabolism and transcription factors.** The expression of *IAMT1*, *YUC3*, *PRE1*, *PRE5*, *PAP1*, and *HAT7* was monitored by RT-qPCR and normalized to the corresponding controls. Values are log ratios between the treatment and the control. PAC, fold change between 0.2 αM PAC- and mock-treated wild type L*er* seedlings; *gai1-ox*, fold change between transgenic and wild type Col-0 seedlings; *rga-*α*17*, fold change between *ProRGA:GFP-(rga-*α*17)* and wild type L*er* seedlings; *gai/rga* null M, fold change between *gai-t6 rga-24* and wild type L*er* seedlings; *gai/rga* null PAC, fold change between PAC-treated and mock-treated *gai-t6 rga-24* seedlings. Three-day-old, dark-grown seedlings of the different genotypes were used. Data represent mean and standard error of the mean from three independent biological replicates. Data from each biological replicate consisted in three technical replicates that were averaged and normalized.
(TIF)

Table S1 **GAI regulated genes in etiolated seedlings.**
(XLS)

Table S2 **List of oligonucleotides used for RT-qPCR.**
(XLS)
