# Supplementary figures and images for: DELLA-Induced Early Transcriptional Changes during Etiolated Development in Arabidopsis thaliana
Source: PLoS One. 2011 Aug 31;6(8):e23918. doi: 10.1371/journal.pone.0023918 (PMC3164146; doi:10.1371/journal.pone.0023918)

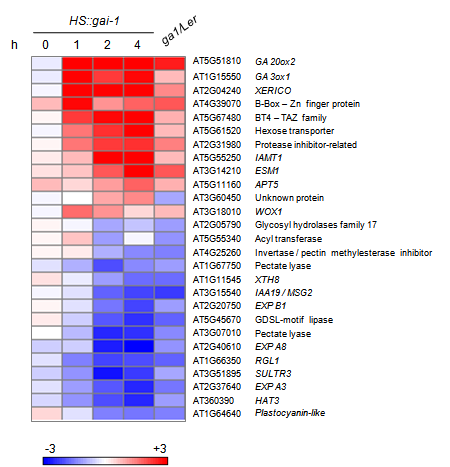

Supplement: Figure S1 — Meta-analysis comparing microarray data from HS::gai-1 and ga1-3 seedlings. Heatmap representation of the differential expression of genes overlapping between the HS::gai-1 and the ga1-3 datasets. Red and blue colors in the heatmaps represent induced and repressed genes, respectively. (TIF) [file pone.0023918.s001.tif]

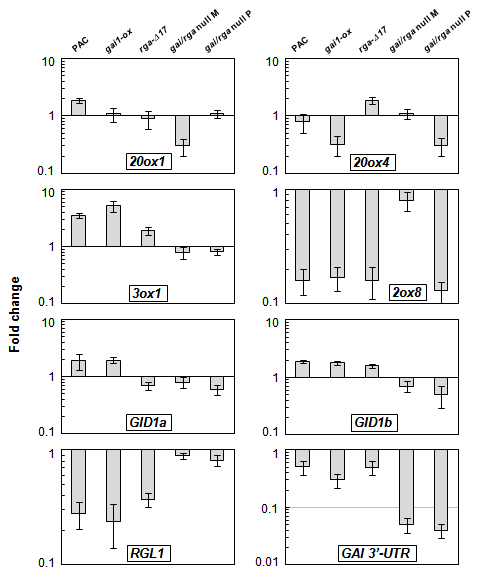

Supplement: Figure S2 — DELLA regulation of GA homeostasis. The expression of genes of the GA pathway was monitored by RT-qPCR and normalized to the corresponding controls. Values are log ratios between the treatment and the control. PAC, fold change between 0.2 αM PAC- and mock-treated wild type Ler seedlings; gai1-ox, fold change between transgenic and wild type Col-0 seedlings; rga-α17, fold change between ProRGA:GFP-(rga-α17) and wild type Ler seedlings; gai/rga null M, fold change between gai-t6 rga-24 and wild type Ler seedlings; gai/rga null P, fold change between PAC-treated and mock-treated gai-t6 rga-24 seedlings. Three-day-old, dark-grown seedlings of the different genotypes were used. Data represent mean and standard error of the mean from three independent biological replicates. Data from each biological replicate consisted in three technical replicates that were averaged and normalized. (TIF) [file pone.0023918.s002.tif]

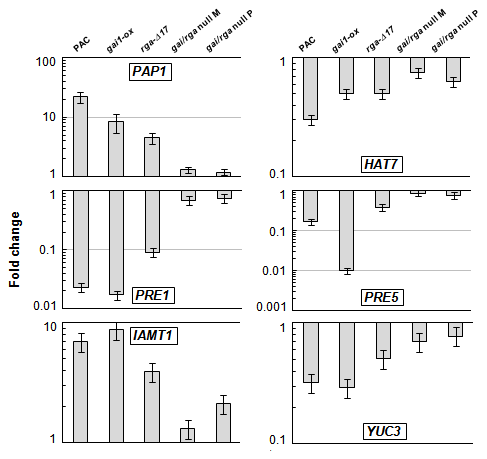

Supplement: Figure S3 — DELLAs regulate the expression of genes of the auxin metabolism and transcription factors. The expression of IAMT1, YUC3, PRE1, PRE5, PAP1, and HAT7 was monitored by RT-qPCR and normalized to the corresponding controls. Values are log ratios between the treatment and the control. PAC, fold change between 0.2 αM PAC- and mock-treated wild type Ler seedlings; gai1-ox, fold change between transgenic and wild type Col-0 seedlings; rga-α17, fold change between ProRGA:GFP-(rga-α17) and wild type Ler seedlings; gai/rga null M, fold change between gai-t6 rga-24 and wild type Ler seedlings; gai/rga null PAC, fold change between PAC-treated and mock-treated gai-t6 rga-24 seedlings. Three-day-old, dark-grown seedlings of the different genotypes were used. Data represent mean and standard error of the mean from three independent biological replicates. Data from each biological replicate consisted in three technical replicates that were averaged and normalized. (TIF) [file pone.0023918.s003.tif]
